# Genomic Convergence Analysis of Schizophrenia: mRNA Sequencing Reveals Altered Synaptic Vesicular Transport in Post-Mortem Cerebellum

**DOI:** 10.1371/journal.pone.0003625

**Published:** 2008-11-05

**Authors:** Joann Mudge, Neil A. Miller, Irina Khrebtukova, Ingrid E. Lindquist, Gregory D. May, Jim J. Huntley, Shujun Luo, Lu Zhang, Jennifer C. van Velkinburgh, Andrew D. Farmer, Sharon Lewis, William D. Beavis, Faye D. Schilkey, Selene M. Virk, C. Forrest Black, M. Kathy Myers, Lar C. Mader, Ray J. Langley, John P. Utsey, Ryan W. Kim, Rosalinda C. Roberts, Sat Kirpal Khalsa, Meredith Garcia, Victoria Ambriz-Griffith, Richard Harlan, Wendy Czika, Stanton Martin, Russell D. Wolfinger, Nora I. Perrone-Bizzozero, Gary P. Schroth, Stephen F. Kingsmore

**Affiliations:** 1 National Center for Genome Resources, Santa Fe, New Mexico, United States of America; 2 Illumina Inc., Hayward, California, United States of America; 3 Department of Neurosciences, University of New Mexico, Albuquerque, New Mexico, United States of America; 4 Northern New Mexico College, Española, New Mexico, United States of America; 5 Department of Psychiatry, University of Alabama at Birmingham, Birmingham, Alabama, United States of America; 6 SAS Institute, Cary, North Carolina, United States of America; Texas A&M University, United States of America

## Abstract

Schizophrenia (SCZ) is a common, disabling mental illness with high heritability but complex, poorly understood genetic etiology. As the first phase of a genomic convergence analysis of SCZ, we generated 16.7 billion nucleotides of short read, shotgun sequences of cDNA from post-mortem cerebellar cortices of 14 patients and six, matched controls. A rigorous analysis pipeline was developed for analysis of digital gene expression studies. Sequences aligned to approximately 33,200 transcripts in each sample, with average coverage of 450 reads per gene. Following adjustments for confounding clinical, sample and experimental sources of variation, 215 genes differed significantly in expression between cases and controls. Golgi apparatus, vesicular transport, membrane association, Zinc binding and regulation of transcription were over-represented among differentially expressed genes. Twenty three genes with altered expression and involvement in presynaptic vesicular transport, Golgi function and GABAergic neurotransmission define a unifying molecular hypothesis for dysfunction in cerebellar cortex in SCZ.

## Introduction

Schizophrenia (SCZ [MIM #181500]) is a common, severe psychiatric disorder with a strong genetic component and complex inheritance [1]–[5]. Cytogenetic, linkage, positional cloning, candidate gene association and genome-wide studies have identified several credible SCZ risk genes. However, few have yet been replicated or translated into causal alleles, *in vitro* diagnostics or therapeutics [6]–[8]. In many studies of SCZ, genetic analysis has been impeded by phenotypic definition based upon multiple, subjectively ascertained, behavioral parameters that lack reference to biological mechanism [9]–[12]. In addition, more than 20 whole-genome linkage scans have demonstrated heterogeneity of linkage [13], suggesting the existence of genocopies (similar phenotypes that are determined by distinct risk loci) [12]. Evidence exists that the genetic architecture of SCZ may be further obscured by allelic heterogeneity (additive genetic variance segregating in the population at causative loci), epistasis (different combinations of loci producing a phenotype in different pedigrees), pleiotropy (loci that affect more than one phenotype) and phenocopies (heterogeneous environmental factors that mimic allelic effects) [14]. In addition, contributions of risk alleles to complex traits may not fit basal multiplicative and/or threshold models, and studies performed to date may not have had sufficient power, or appropriate theory, to assess non-linear (*i.e.*, epistatic and genotype-by-environment) models. As a result, case-control association studies have identified numerous significantly associated susceptibility loci, but lack of replication among studies is widespread [3], [4], [8], [15]–[18]. Furthermore, the most validated loci were largely selected based on involvement in networks implicated in SCZ (such as dopaminergic and glutamatergic neurotransmission), introducing bias and limiting identification of novel risk factors.

Absence of a clear understanding of the molecular basis of SCZ imposes significant challenges to timely diagnosis and prognostic or therapeutic categorization [12], [19]. Supplementation of diagnostic criteria with biomarkers that are causally related to SCZ or endophenotypes may allow definition of homogeneous subgroups that are predictive of progression and therapeutic response in individual patients [20] and would serve as a starting point for development of therapeutics directed at causal variants. An alternative approach for molecular dissection of SCZ is identification of altered gene expression in affected tissues. Because gene expression reflects both genetic and environmental influences, it may be particularly useful for identifying risk factors for a complex disorder such as SCZ, which is believed to have a multifactorial etiology [21]. Two factors have hitherto limited the effectiveness of gene expression analysis in SCZ: Firstly, mRNA analyses in post-mortem brains in SCZ is challenging due to type I and type II errors resulting from variation in cause of death (affecting agonal gene expression), postmortem interval (affecting RNA quality), concurrent medication, substance abuse, age, sex, race and duration of illness [22]. Secondly, in common with genome-wide association studies, gene expression comparisons employing available cohort sizes sail between the Scylla of many false-positives due to multiple comparisons and the Charybdis of insufficient power to detect true-positives following statistical correction [23]. Recently, however, studies of mRNA expression in post mortem brains in SCZ that account for these variables have started to be reported [7], [21], [22].

An elegant, new approach to navigate Scylla and Charybdis and, thereby, accomplish molecular definition of SCZ may be genomic convergence analysis [24]. Predicated on an implication of the central dogma of molecular biology, genomic convergence analysis posits that clinically relevant nucleotide variation should result in detectable *cis*- and *trans*-effects in messenger RNA (mRNA) that amalgamate into functional changes in networks and pathways. Importantly, genomic convergence analysis provides a strategy to collectively interpret and employ the massive, disease-related data sets produced by unbiased (i.e. non-hypothesis driven) linkage and expression studies. Indeed, integration of gene expression and genetic linkage data has shown promise in several neurologic disorders [24]–[27] and has started to be applied to SCZ [7], [28]–[30].

mRNA sequencing with shotgun, massively parallel sequencing platforms has recently shown utility for measurement of transcript abundance, splice isoforms and allelic influence on gene expression [31]–[50]. mRNA abundance is determined by sequencing either 3′ end tags or random cDNA fragments (digital transcript expression, DTE), followed by read alignment to reference databases and calculation of aligned read frequencies. Potential advantages of DTE in comparison to array hybridization include: single molecule sensitivity (corresponding to approximately 1 mRNA molecule per 30 cells; Hayashizake, personal communication); absolute, rather than relative, measurement of transcript abundance; sequence verification for each measurement; comprehensive detection of both known and novel, unannotated transcripts and isoforms; applicability to any eukaryotic species; very little technical imprecision; absence of interference from abundant transcripts (e.g. globin); and extensibility to concomitant measurement of non-coding RNA and to detection of nucleotide and structural variation.

As the first stage of a genomic convergence analysis of SCZ, we describe shotgun mRNA sequencing of an affected tissue (post-mortem cerebellar cortex) of patients and controls, together with a rigorous and systematic approach to DTE. The approach presented is conservative due to application of statistical and bioinformatic methods that substantially reduce type I error rates by using statistical significance criteria rather than fold-change values and by incorporation of potential confounding variables, such as psychotropic medication and cause of death. It is also more comprehensive than prior microarray studies of SCZ. The stringency of this approach, in combination with genome-wide genotypes from the same individuals, is anticipated to enable a genomic convergence analysis that advances understanding of the molecular basis of SCZ.

## Results

### Sequencing-by-synthesis of cerebellar mRNA

**Figure 1:**
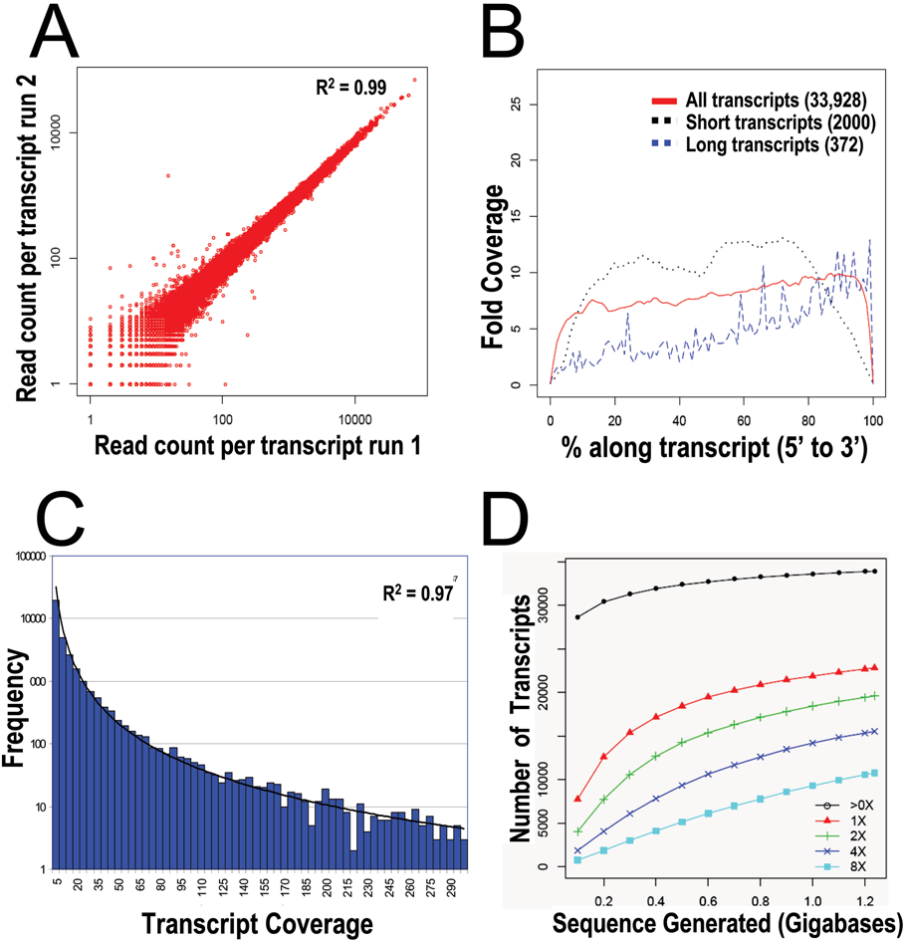
Characteristics of cDNA sequencing-by-synthesis with Illumina/Solexa instrument. (A) Run-to-run comparisons of the number of reads aligned per reference transcript for 2 SBS runs of cerebellar cortex #41 (21.6 and 17.0 million reads, respectively). Runs used the same library but were performed on different sequencing instruments on different days. Coefficient of variation was 3.4%. (B) Histogram showing coverage along an “averaged” reference transcript for 1.2 Gb of cerebellar cortex #41 cDNA sequences. Coverage was calculated at 1% intervals along each transcript to which reads aligned. At each interval coverage was averaged across all transcripts and plotted. “Short transcripts” are all transcripts of ≤500 bp to which reads were aligned. “Long transcripts” are all transcripts ≥10 kb to which reads were aligned. Numbers in parentheses are the number of transcripts represented by each category. (C) Histogram showing greater than exponential decline in frequency with increasing SBS coverage on reference transcripts for cerebellar cortex #41 (1.2 Gb). Of 33,938 transcripts, 111 had more than 300× coverage. Maximum coverage was 2323×. Best fit power trendline is shown. (D) Histogram showing the number of reference transcripts covered as a function of the amount of sequence generated. Different levels of minimum average coverage were examined: at least one read aligned (black circles), 1× average coverage (red triangles), 2× average coverage (green “+”), 4× average coverage (dark blue “×”), and 8× average coverage (light blue squares). Data are from SBS of cerebellar cortex #41 (1.2 Gb).

16.7 billion nucleotides of shotgun, full length cDNA sequence data were generated using Illumina Genome Analyzer platforms with sequencing-by-synthesis (SBS) chemistry from 20 mRNA samples [39], [42], [43]. mRNA samples were isolated post-mortem from the lateral hemispheres of the cerebellar cortices of 14 patients with SCZ and 6 control individuals [51], [52] (Table 1). Unrelated subjects were chosen to facilitate sampling of genetic heterogeneity [53], [54]. Cases and controls were approximately matched for age (cases, 45.2±11.8 years; controls 41.3±9.2 years), sex (all male), race, post-mortem interval (cases, 12.2±5.0 hours; controls 17.7±3.3 hours), cause of death, autopsy brain pH (cases, 6.54±0.19; controls 6.46±0.10) and RNA integrity number (cases, 8.06±0.53; controls, 7.87±0.41) (Table 1). 12.5–38.7 million, high quality sequences of length 32–36 bp were generated per sample (Table 2). Sequences were aligned to the human genome and RefSeq transcript databases using the algorithm GMAP, which allowed <2 (≤6%) mismatches [55]. There was little intra-sample variability in the number of sequences aligned to each locus from run-to-run or instrument-to-instrument (Fig. 1A; all source coefficient of variation 3.4%). 43.5±6.7% of sequences aligned to a transcript and 69.4±9.6% to the genome, evidence that annotation of mRNA isoforms in Homo sapiens is incomplete [56] (Table 2). 91% of alignments were unique (Table 2). Reads aligning to more than one location contained repetitive, paralogous, polymorphic or low complexity sequences and primarily mapped to untranslated regions or highly polymorphic gene families, such as major histocompatibility genes [48]. Unmapped sequences did not align to mitochondrial or 1879 viral genomes, offering negative evidence of chronic viral etiology for SCZ in these patients.

**Table 1:** Clinical Features of Patients and mRNA Samples.

Sample	Diagnosis	Age (Years)	Race	Death Cause	Treatment	Post Mortem Interval	Brain pH	RIN^1^
1	SCZD	62	African American	ASCVD^2^	Olanzapine	12 hours	6.56	7.4
11S	SCZD	38	African American	Pneumonia	Chlorpromazine	5 hours	6.64	8.5
18	SCZD	45	Caucasian	Suicide	Olanzapine	6 hours	6.65	8.4
1S	SCZD	53	Caucasian	ASCVD	Perphenazine	11 hours	6.69	7.9
2	SCZD	69	African American	ASCVD	Haloperidol	14 hours	6.71	8.2
31	SCZD	46	Caucasian	ASCVD	Olanzapine	20 hours	6.42	9.3
36	SCZD	59	Caucasian	Embolism	Haloperidol	13 hours	6.67	8.4
39	SCZD	31	African American	ASCVD	Clozapine	14 hours	6.74	8.3
3S	SCZD	38	African American	Asphyxia	Prolixin	6 hours	6.22	7.8
41	SCZD	37	Caucasian	Suicide	Risperidone	14 hours	6.68	7.3
42	SCZD	33	African American	Apendicitis	Olanzapine	12 hours	6.21	7.4
5	SCZD	49	Caucasian	Intoxication	Thioridazine	16 hours	6.55	8.1
5S	SCZD	42	African American	Tuberculosis	Trilafon	21 hours	6.25	7.8
7S	SCZD	32	Caucasian	Suicide	Clozapine	7 hours	6.58	8.0
17	Control	48	Caucasian	SAH	.	19 hours	6.51	8.0
2S	Control	50	Caucasian	ASCVD	.	22 hours	6.38	7.8
35	Control	33	African American	Obesity	.	14 hours	6.50	8.6
40	Control	43	Caucasian	ASCVD	.	20 hours	6.29	7.5
6S	Control	47	Caucasian	Arrhythmia	.	17 hours	6.52	7.5
8S	Control	27	African American	Asthma	.	14 hours	6.56	7.8

^1^RNA Integity Number; ^2^Arteriosclerotic Cardiovascular Disease.

**Table 2:** cDNA Sequencing and Alignment Statistics.

Sample	Year Sequenced	Average Read Length	Average Read Quality^3^	Number of reads	Reads Aligning to Genome^2^	Unique Genome Alignments	Reads Algining to Transcripts^1^	Unique Transcript Alignments	Transcript Matches (1000 s)	Average Transcript Coverage
1	2007	32	20	23,241,938	78%	68%	50%	45%	33.3	9.4×
11S	2007	35	28	14,572,861	65%	54%	44%	37%	31.4	7.0×
18	2007	32	21	25,129,004	79%	69%	52%	46%	33.8	9.6×
1S	2007	32	19	36,760,977	73%	64%	48%	43%	34.6	12.7×
2	2008	36	31	19,241,726	69%	60%	36%	32%	33.4	6.2×
31	2008	36	33	19,867,823	71%	63%	40%	36%	32.9	6.8×
36	2007	32	21	20,111,871	77%	67%	46%	41%	33.0	7.0×
39	2007	33	29	23,055,778	72%	63%	44%	39%	34.8	8.3×
3S	2007	34	26	17,846,750	52%	46%	35%	31%	32.4	5.8×
41	2007	32	21	38,658,913	75%	66%	50%	45%	33.9	13.4×
42	2007	35	28	17,588,723	63%	56%	38%	34%	33.7	5.5×
5	2008	36	31	21,229,299	70%	61%	37%	33%	32.9	7.5×
5S	2007	32	21	28,944,566	77%	66%	42%	36%	34.0	10.0×
7S	2007	34	25	13,769,073	61%	54%	40%	36%	32.1	4.7×
17	2008	36	22	12,890,252	52%	47%	35%	31%	31.5	4.0×
2S	2008	36	23	12,482,759	49%	44%	31%	28%	31.4	3.4×
35	2008	36	27	25,402,905	71%	63%	48%	44%	33.8	10.3×
40	2008	36	27	24,486,091	72%	64%	47%	42%	33.4	9.7×
6S	2008	32	23	24,347,196	80%	71%	54%	48%	33.4	9.4×
8S	2008	32	22	24,016,465	81%	71%	52%	46%	33.5	9.4×

^1^Alignments to RefSeq Human transcript database, Release 22; ^2^Alignments to NCBI Human genome sequence, Build 36.2; ^3^Uncalibrated quality scores.

In order to further investigate differences in proportions of sequences aligning to different reference databases, an additional 2.2 billion nucleotides of shotgun, full length, paired cDNA sequence data were generated from mRNA sample 3S using an Illumina Genome Analyzer II platform and aligned to three reference databases (the human genome, RefSeq transcript and UniGene transcript databases): 46.1% of reads aligned to all three databases, representing known exons. 31.4% of reads aligned to the genome alone, representing novel exons, splice isoforms [57] or transcripts [56]. 5.5% mapped to RefSeq transcript alone, and 6.5% to UniGene transcript alone, representing sequences that span known exon junctions [57]. 10.6% of reads did not align to any of these reference sets, representing reads that span boundaries of novel exons or splice isoforms, novel genomic sequence, or poor quality sequence.

### Coverage and Composition of cDNA Sequences

The number of transcripts detected in cerebellar cortex mRNA differed little between samples (33,200±1,000; Table 2), corresponding to 85±3% of RefSeq transcript entries. While 12.5 million sequences per sample was sufficient to reach a plateau in the number of transcripts detected, deeper sequence generation resulted in linear increase in average depth of coverage (Fig. 1D). As anticipated with hexamer-primed cDNA synthesis, the distribution of sequence alignments along transcripts appeared random. Transcript coverage showed a moderate 3′ bias, particularly among long transcripts, attributable to only very slight levels of degradation in these samples together with two rounds of poly-A^+^ RNA selection (Fig. 1B). Decreased coverage was observed at 5′ and 3′ termini due to edge effects of random priming very close (<30 nts) to the ends of the mRNA. No compositional bias was detected. The average depth of coverage achieved was 8.0-fold (Table 2). The distribution of transcript abundance in cerebellar cortex was interesting: 76–81% of expressed transcripts had an abundance of at least 1 read per million. However, as abundance increased, the number of transcripts declined greater than exponentially (Fig. 1C, r^2^ = 0.97). Thus, only 0.3–0.5% of expressed transcripts had an abundance of ≥1000 reads per million.

### Comparison of Digital Transcript Expression and Array Hybridization

**Figure 2:**
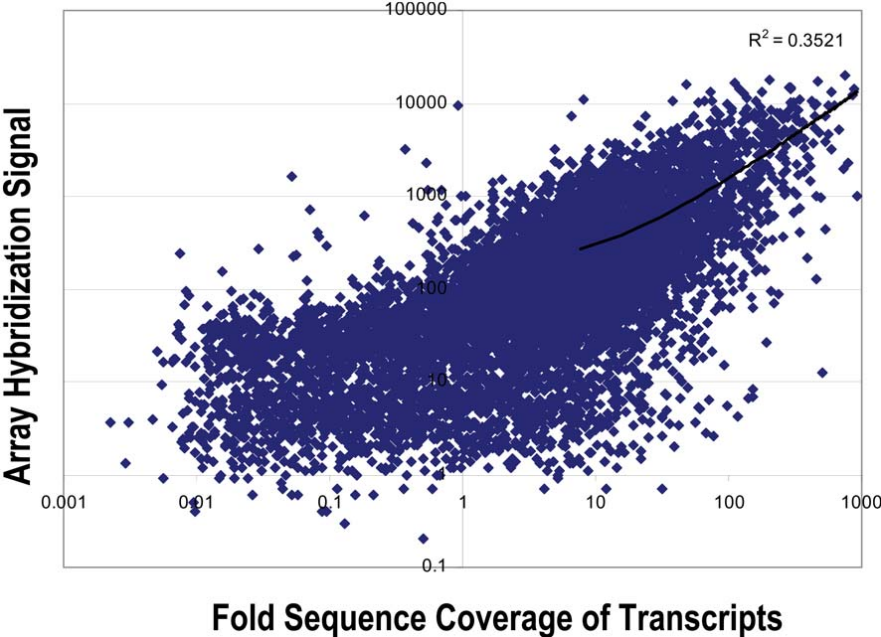
Comparison of gene expression levels measured by two methods. Comparison of gene expression levels detected by average shotgun, mRNA SBS coverage per transcript (normalized for transcript length) versus Affymetrix oligonucleotide microarray hybridization for cerebellar cortex sample #41 (38.7 million reads). Microarray images were scaled to an average hybridization intensity of 200, and the threshold for expression was an average hybridization intensity of ≥50.

**Figure 3:**
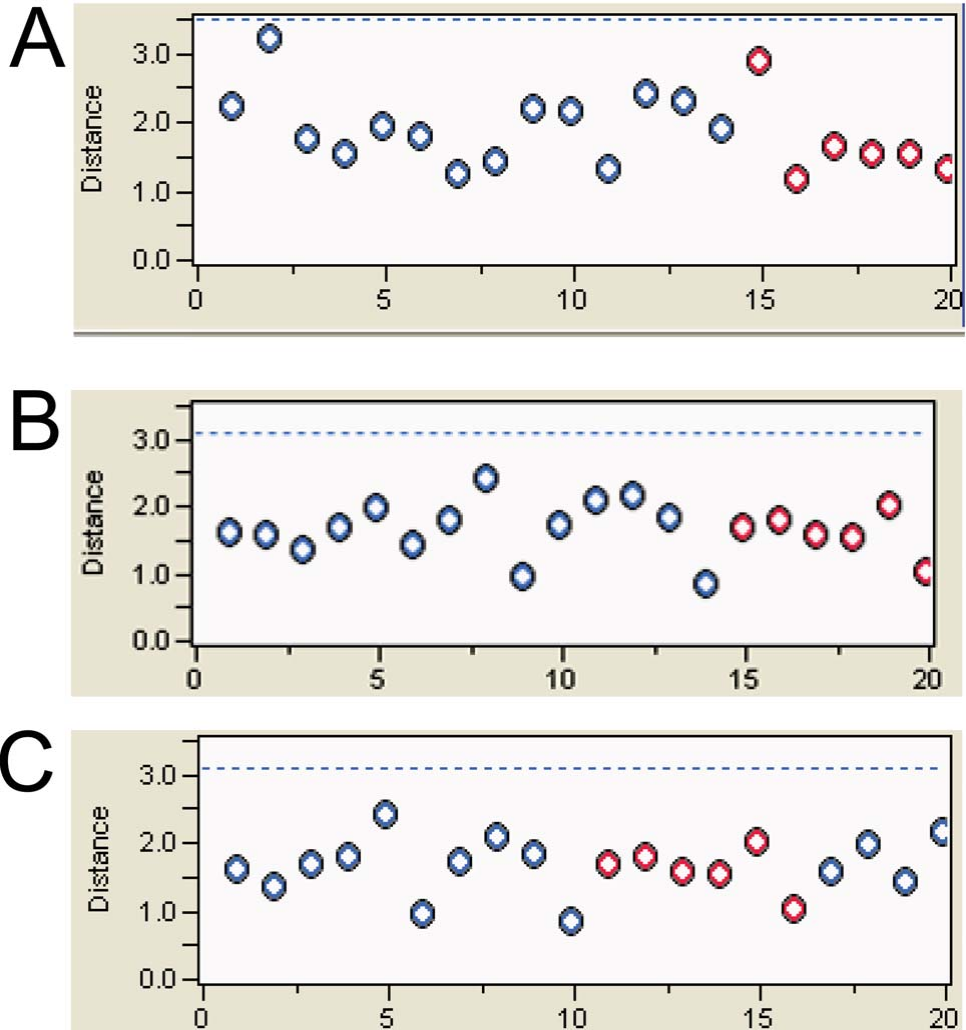
Comparison of Mahalanobis Distances of Gene Expression by Array Hybridization and Sequence Read Frequencies. 14 SCZ samples are indicated by blue circles, and 6 control samples by red circles. The Y-axis shows Mahalanobis distances of log transformed gene expression values. The dotted blue line indicates the cutoff value for outliers. Panel A: Log10 transformed Affymetrix array hybridization signals. Panel B: Log10 transformed genome-aligned read frequencies. Panel C: Log10 transformed transcript-aligned read frequencies. Log10 transformed array hybridization values (A) had a wider distribution of distances than Log10 transformed sequence read frequencies (B,C). Without log transformation, distances were greater and several samples represented outliers (data not shown).

**Figure 4:**
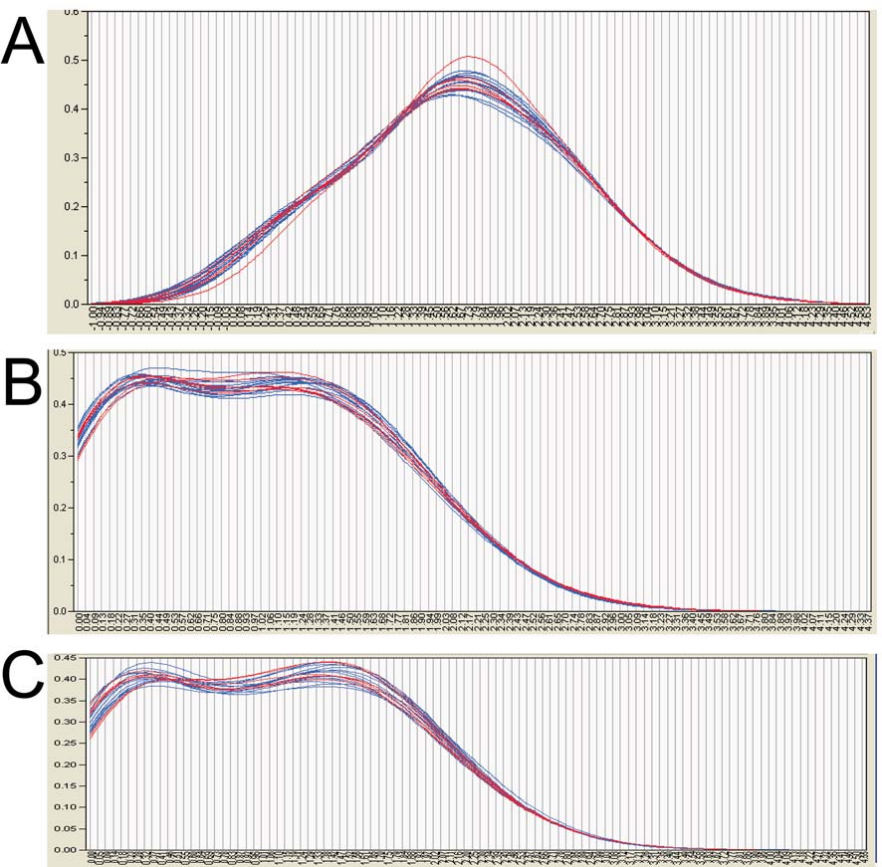
Overlayed kernel density estimates of Gene Expression by Array Hybridization and Sequence Read Frequencies. 14 SCZ samples are indicated by blue lines, and 6 control samples by red lines. The X-axis shows log transformed gene expression values while the Y-axis shows kernel densities. Panel A: Log10 transformed Affymetrix array hybridization signals. Panel B: Log10 transformed genome-aligned read frequencies. Panel C: Log10 transformed transcript-aligned read frequencies. Log10 transformed array hybridization values less than 1.69 (equivalent to a calibrated hybridization signal of 50) are considered noise. Without log transformation, samples showed greater variability in kernel densities and sequence read frequencies showed a near exponential decay (Fig. 1C, data not shown).

**Figure 5 pone-0003625-g005:**
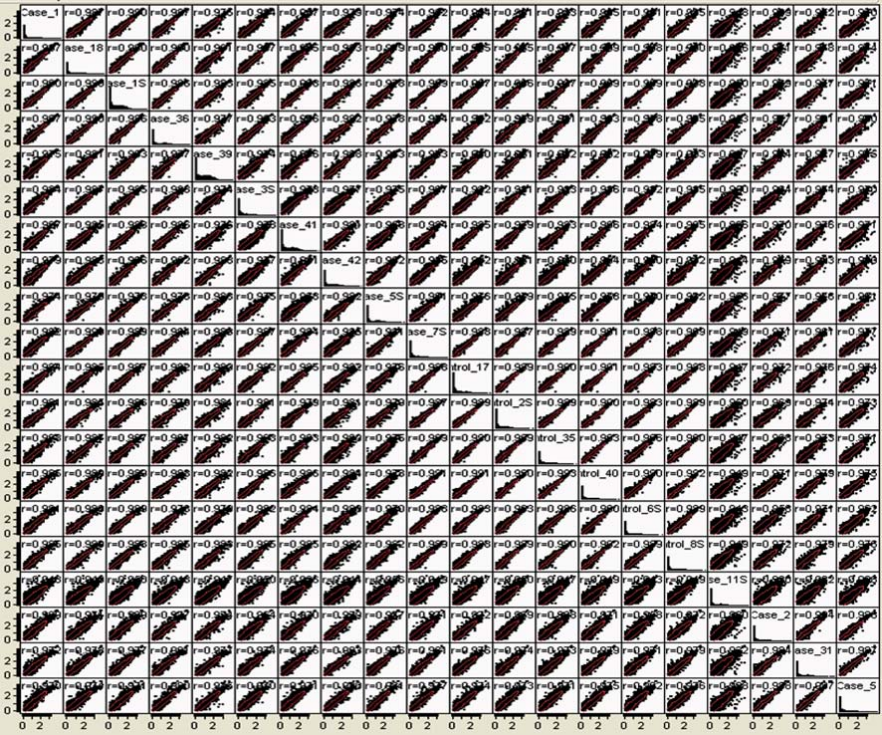
Pairwise sample correlations of log10-transformed, genome-aligned read frequencies, showing pairwise correlation coefficients.

**Figure 6 pone-0003625-g006:**
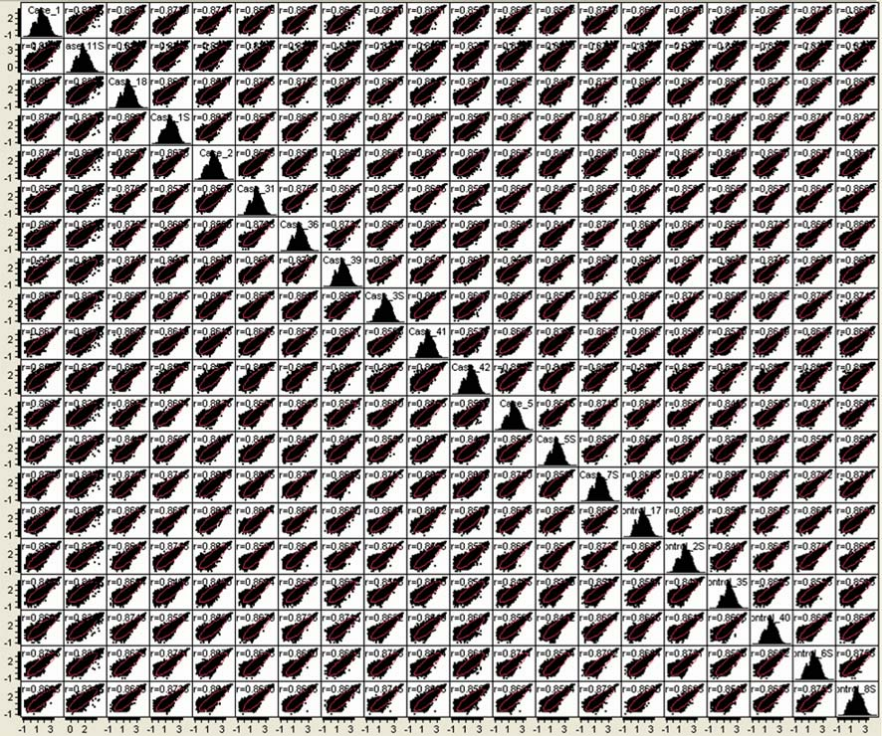
Pairwise sample correlations of log10-transformed, array hybridization signals, showing pairwise correlation coefficients.

**Figure 7 pone-0003625-g007:**
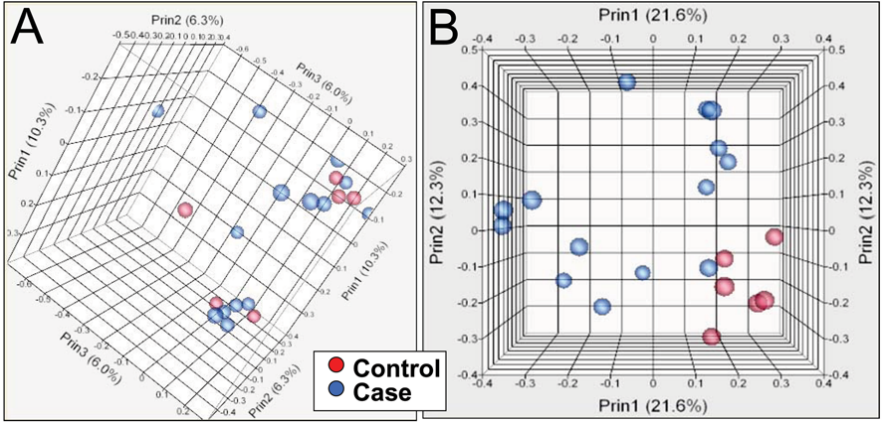
Unsupervised principal component analysis of log10 transformed array hybridization signals (A) and log10 transformed read frequencies (B). Three dimensional plots of principal component analysis by Pearson product-moment correlation. 14 SCZ samples are indicated by blue circles, and 6 control samples by red circles.

Gene expression was evaluated in mRNA samples from the 20 cerebellar cortices using both oligonucleotide array hybridization, the current standard, and tag frequencies from the Illumina cDNA sequencing assay (aligned reads per million). The dynamic range of read frequencies was two orders of magnitude greater than array hybridization (log_10_ dynamic range DTE–4.37; log_10_ dynamic range array hybridization–2.33; Fig. 2). While 41% of arrayed oligonucleotide probes had hybridization intensities greater than the conventional signal: noise threshold (Fig. 2), 85±3% transcripts had aligned reads. Array hybridization signals are transformed and often normalized prior to evaluation of inter-sample differences. For read frequencies, log transformation, but not normalization, improved overlaid kernel density estimates, univariate distribution results, and Mahalanobis distances (Fig. 3 and 4). Therefore, log transformed values were used in all subsequent expression analyses. Aligned read frequencies, adjusted for transcript length, correlated weakly with oligonucleotide array hybridization signals (r^2^ = 0.35; Fig. 2). Read frequencies exhibited much higher correlation coefficients in pair wise sample comparisons (r^2^ = 0.93–0.99) than array hybridization (r^2^ = 0.83–0.88; Fig. 5 and 6). Furthermore, pair wise sample correlations of genome- and transcript-aligned read frequencies had very similar correlation coefficients (data not shown). SCZ patients could readily be distinguished from controls by unsupervised principal component analysis (by Pearson product-moment correlation, Fig. 7B) or Ward hierarchical clustering of Pearson product-moment correlations of read frequencies (data not shown). In contrast, patients and controls could not be separated with these methods on the basis of array hybridization signals (Fig. 7A and data not shown). Log transformed genome- and transcript-aligned read frequencies showed identical hierarchical clustering and Pearson product-moment correlations of samples (data not shown).

**Figure 8 pone-0003625-g008:**
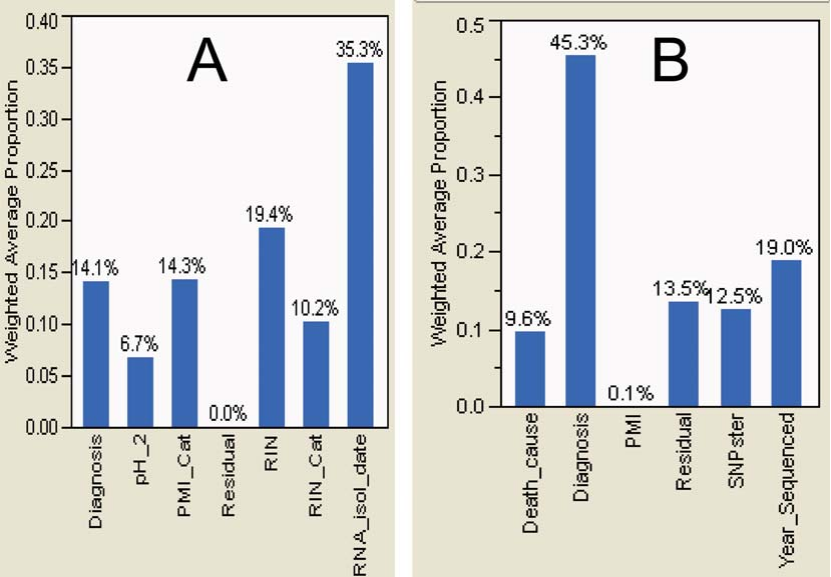
Principal components of variance of log10 transformed array hybridization signals (A) and log10 transformed read frequencies (B). Variance components decomposition of principal components (with Pearson correlation), with partitioning of variability in terms of known effects. Patient (Diagnosis, Cause of death [Death_cause], Age, Race, Medication), sample (Post-mortem interval [PMI], brain pH [pH_2], RNA integrity number [RIN], RNA isolation date [RNA_isol_date]), and experimental (Year Sequenced, Average Read Quality, Average Read length, Cluster Station, Illumina 1G instrument [SNPster], Library Creator, % reads aligning, number of reads) parameters were examined to quantify sources of variability in read-frequency- and array hybridization-based gene expression.

Patient, sample, and experimental parameters were examined to quantify sources of variability in read-frequency-based DTE. Decomposition of principal components of variance showed that DTE attributed a greater proportion of the total variance to diagnosis (SCZ versus control, 45.3%) than array hybridization (14.1%) (Fig 8). The largest sources of variability of array hybridization results were mRNA quality metrics (Fig. 8A), whereas DTE results were only marginally influenced by mRNA quality. In contrast, large sources of variability in DTE results were year of sequence generation, sequencing instrument-to-instrument variation and cause of death (Fig. 8B). Variance component categories were identical in genome- and transcript-aligned read frequencies (data not shown).

### Digital Transcript Expression Results

**Figure 9:**
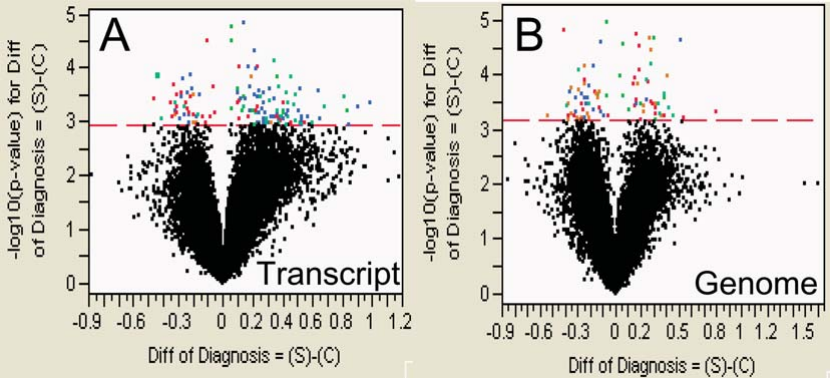
Volcano plot of analysis of variance of log10-transformed, transcript- (A) and genome-aligned read frequencies (B), showing genes with FDR-corrected differences in LSMeans. The x-axis shows the magnitude of the difference between 14 cases (S) and 6 controls (C), while the Y-axis shows the −log10(p value) for those differences. The red dashed line indicates the significance cutoff for differences with a control (−log10(p value) 3.17 for genome alignments and 2.94 for transcript alignments).

Differences between SCZ patients and controls in gene expression in cerebellar cortex were identified with analysis of variance with diagnosis as the discriminatory effect and the major non-diagnosis components of variance as fixed effects (brain pH, post-mortem interval, RNA integrity number and RNA isolation date for array hybridization, and cause of death, post-mortem interval, sequencing instrument and year sequenced for read frequencies). Following FDR correction, no differences were identified between SCZ patients and controls by array hybridization, due to the magnitude of non-diagnosis components of variance. In contrast, 88 genes exhibited FDR-corrected differences in cerebellar expression between SCZ patients and controls in genome-aligned read frequencies and 152 genes differed significantly in transcript-aligned reads (Fig. 9; Table 3). Between these two sets of genes, 25 were identified in common to both genome- and transcript-aligned reads. 24 of these 25 genes showed congruent direction of change in genome- and transcript-aligned reads. 95% of genes exhibiting significant change in expression with only one alignment had congruent but non-significant change in the second alignment or were absent in one of the reference sets. The correlation coefficient of the case-control ratio of the 215 differentially expressed genes between genome- and transcript-aligned reads was 0.73 and between transcript-aligned reads and array hybridization was 0.46 (data not shown). A majority of genes with disparity in the magnitude of gene expression change between alignments had greater read counts for genome- than transcript-alignments, which, in some cases, appeared to be due to unannotated exons and novel splice isoforms (see below).

**Table 3:** Genes Exhibiting Significantly Altered Aligned Read Frequencies between SCZD Cases and Controls.

Gene	Gene	Golgi or	Transcript^1^	Genomic	Transcript	Genomic
Symbol	Name	Vesicular	Alignment	Alignment	Alignment	Alignment
		Transport	Expression	Expression	Fold	Fold
		Annotation	−log10 p value	−log10 p value	Change	Change
NKX2-3	NK2 transcription factor related, locus 3 (Drosophila)	No	4.49	0.74	large^2^	3.20
SCGB3A2	secretoglobin, family 3A, member 2	No	3.23	0.44	large	0.71
C10orf136	chromosome 10 open reading frame 136	No	3.21	0.87	large	0.49
TMIGD1	transmembrane and immunoglobulin domain containing 1	No	0.95	3.34	large	0.12
FAM55A	family with sequence similarity 55, member A	No	3.17	1.76	large	0.07
NR1H4	nuclear receptor subfamily 1, group H, member 4	No	4.76	0.27	large	−0.10
MAGEC2	melanoma antigen family C, 2	No	4.49	0.33	large	absent^3^
ISL2	ISL2 transcription factor, LIM/homeodomain	No	3.60	0.37	8.80	8.07
AMMECR1	AMME Complex, gene 1	No	2.97	2.35	5.80	0.63
HMGCS2	3-hydroxy-3-methylglutaryl-Coenzyme A synthase 2 (mitochondrial)	No	3.84	0.74	3.72	0.53
KLHDC1	kelch domain containing 1	No	2.94	0.87	3.60	0.03
IMPG2	interphotoreceptor matrix proteoglycan 2	No	1.30	4.67	3.21	0.52
C14orf168	dynein, axonemal, light chain 1	No	3.20	0.71	2.82	0.16
SGOL2	shugoshin-like 2	No	3.17	1.28	2.56	0.42
LOC400986	protein immuno-reactive with anti-PTH polyclonal antibodies	No	3.26	2.96	2.32	0.90
KRT6E	keratin 6C	No	4.76	0.13	2.05	−0.61
MAN1A2	mannosidase, alpha, class 1A, member 2	No	3.34	3.34	2.00	1.26
LOC649841	similar to protein immuno-reactive with anti-PTH polyclonal antibodies	No	3.47	absent	1.98	absent
RIF1	RAP1 interacting factor homolog	No	3.03	2.08	1.80	0.67
CACNG2	calcium channel, voltage-dependent, gamma subunit 2	No	3.05	1.00	1.69	0.10
C6orf204	chromosome 6 open reading frame 204	No	3.27	1.77	1.41	0.29
LOC644734	similar to Zinc finger protein 85 (Zinc finger protein HPF4) (HTF1)	No	3.08	1.03	1.30	0.13
TMTC3	transmembrane and tetratricopeptide repeat containing 3	No	3.30	1.78	1.26	0.71
SNORA76	small nucleolar RNA, H/ACA box 76	No	4.50	0.78	1.22	0.45
KIAA1212	KIAA1212	No	3.56	3.21	1.19	0.84
ALG10	asparagine-linked glycosylation 10 homolog	No	2.35	3.46	1.05	1.62
ARHGAP5	Rho GTPase activating protein 5	No	2.83	3.26	1.01	0.69
LOC731295	similar to microtubule associated serine/threonine kinase 2	No	3.64	absent	1.00	absent
LOC729937	similar to microtubule associated serine/threonine kinase 2	No	3.18	1.20	0.96	1.42
ZNF148	zinc finger protein 148	No	2.98	1.81	0.93	0.21
TCP10L	t-complex 10 (mouse)-like	No	4.14	1.37	0.93	0.28
CLEC4E	C-type lectin domain family 4, member E	No	3.59	0.53	0.93	−0.27
LOC157489	similar to SDA1 domain containing 1	No	3.36	3.57	0.90	0.86
EEA1	early endosome antigen 1, 162 kD	Yes	3.09	1.04	0.88	−0.01
PAMCI	peptidylglycine alpha-amidating monooxygenase COOH-terminal interactor	No	2.95	0.40	0.86	−0.23
BIRC3	baculoviral IAP repeat-containing 3	No	3.08	0.44	0.86	0.30
ROCK1	Rho-associated, coiled-coil containing protein kinase 1	Yes	2.95	2.50	0.85	0.60
LOC728222	hypothetical protein LOC728222	No	2.99	0.19	0.81	0.04
MYO9A	myosin IXA	No	3.20	1.87	0.81	0.53
MYSM1	myb-like, SWIRM and MPN domains 1	No	2.95	2.21	0.79	0.57
ZCRB1	zinc finger CCHC-type and RNA binding motif 1	No	3.32	2.31	0.77	0.59
RB1	retinoblastoma 1 (including osteosarcoma)	No	3.35	3.39	0.74	0.47
LOC732360	similar to G/T mismatch-specific thymine DNA glycosylase	No	3.02	absent	0.71	absent
MARCH7	membrane-associated ring finger (C3HC4) 7	No	1.58	3.86	0.70	0.43
SLC35A3	solute carrier family 35 (UDP-N-acetylglucosamine transporter), member A3	Yes	3.14	1.92	0.69	0.18
WDR67	WD repeat domain 67	No	1.85	3.95	0.68	0.23
B3GALT1	UDP-Gal:betaGlcNAc beta 1,3-galactosyltransferase, polypeptide 1	No	2.24	3.38	0.68	0.77
RBM12B	RNA binding motif protein 12B	No	4.15	4.19	0.67	0.50
LMBRD1	LMBR1 domain containing 1	No	1.75	4.46	0.67	0.32
ZNF534	zinc finger protein 534	No	3.54	1.19	0.65	0.25
NAALAD2	N-acetylated alpha-linked acidic dipeptidase 2	No	2.11	3.90	0.65	0.99
BAAT	glycine N-choloyltransferase	No	3.20	0.91	0.65	0.20
FRS2	fibroblast growth factor receptor substrate 2	No	3.17	1.82	0.64	0.40
AAK1	AP2 associated kinase 1	Yes	3.36	2.08	0.64	0.34
LOC129522	similar to RalA-binding protein 1	No	3.48	2.68	0.62	0.41
TCF4	transcription factor 4	No	2.97	2.02	0.59	0.57
ZNF195	zinc finger protein 195	No	2.97	2.26	0.59	0.29
JMY	junction-mediating and regulatory protein	No	3.06	0.69	0.57	0.55
NFE2L2	nuclear factor (erythroid-derived 2)-like 2	No	3.12	3.27	0.57	0.45
VPS35	vacuolar protein sorting 35 homolog	Yes	2.96	2.96	0.52	0.31
GABRA1	gamma-aminobutyric acid A receptor, alpha 1	No	3.19	3.25	0.52	0.46
KIAA0256	KIAA0256 gene product	No	3.03	3.04	0.51	0.41
SQLE	squalene epoxidase	No	3.11	2.67	0.51	0.39
LOC730549	similar to 60S ribosomal protein L7a	No	3.08	absent	0.50	absent
LOC728315	similar to 60S ribosomal protein L7a	No	3.03	0.40	0.49	0.11
TRIP11	thyroid hormone receptor interactor 11	Yes	3.22	2.72	0.48	0.23
FAM8A1	family with sequence similarity 8, member A1	No	3.58	4.10	0.47	0.33
AFF4	AF4/FMR2 family, member 4	No	3.09	0.33	0.46	−0.03
KIAA0423	KIAA0423	No	3.06	1.92	0.45	0.34
LOC285636	hypothetical protein LOC285636	No	3.58	3.10	0.44	0.38
EIF3S1	eukaryotic translation initiation factor 3, subunit 1 alpha, 35 kDa	No	3.04	2.50	0.43	0.25
SRPK2	SFRS protein kinase 2	No	2.97	2.42	0.42	0.28
PHF14	PHD finger protein 14	No	3.61	2.27	0.40	0.41
LOC647969	similar to basic transcription factor 3	No	3.20	absent	0.39	absent
USP8	ubiquitin specific peptidase 8	No	3.10	2.46	0.39	0.42
LOC163131	zinc finger protein 780B	No	2.20	3.29	0.39	0.54
TBC1D12	TBC1 domain family, member 12	No	2.98	1.69	0.39	0.24
DENND4A	DENN/MADD domain containing 4A	No	3.43	1.97	0.38	0.35
C8orf53	chromosome 8 open reading frame 53	No	3.81	3.29	0.37	0.30
RANBP5	RAN binding protein 5	No	4.00	1.88	0.37	0.28
ST7OT2	ST7 overlapping transcript 2 (antisense RNA)	No	3.18	1.39	0.37	0.28
VDP	vesicle docking protein p115	Yes	3.53	4.54	0.36	0.25
RAB9B	RAB9B	Yes	2.49	3.18	0.35	0.23
DGKE	diacylglycerol kinase, epsilon 64 kDa	No	2.11	3.47	0.35	0.50
LOC283523	similar to telomeric repeat binding factor 1 isoform 2	No	1.20	3.65	0.32	0.97
RBM41	RNA binding motif protein 41	No	2.22	3.24	0.32	0.61
MTRF1L	mitochondrial translational release factor 1-like	No	2.96	2.75	0.31	0.31
COG6	component of oligomeric golgi complex 6	Yes	2.40	3.50	0.30	0.37
KIAA0895	KIAA0895 protein	No	3.64	1.73	0.30	0.31
KIAA1324L	KIAA1324-like	No	3.14	1.55	0.29	0.29
GOLGA1	golgi autoantigen, golgin subfamily a, 1	Yes	4.33	3.84	0.28	0.14
ZNF658	zinc finger protein 658	No	2.99	2.03	0.28	0.12
LASS6	LAG1 homolog, ceramide synthase 6	No	2.59	4.68	0.28	0.40
E2F8	E2F transcription factor 8	No	1.09	3.61	0.27	0.11
MFAP3	microfibrillar-associated protein 3	No	2.91	3.18	0.26	0.25
WWP1	WW domain containing E3 ubiquitin protein ligase 1	No	2.55	3.54	0.26	0.24
ZBTB38	zinc finger and BTB domain containing 38	No	3.35	3.13	0.25	0.21
PLCB4	phospholipase C, beta 4	No	1.55	4.65	0.24	0.95
BTG1	B-cell translocation gene 1, anti-proliferative	No	3.09	2.38	0.24	0.14
SMEK2	SMEK homolog 2, suppressor of mek1 (Dictyostelium)	No	3.14	2.78	0.21	0.22
LOC341315	hypothetical LOC341315	No	3.10	2.48	0.21	0.04
NAP1L3	nucleosome assembly protein 1-like 3	No	3.08	1.22	0.20	0.11
RIT2	Ras-like without CAAX 2	No	0.88	3.99	0.20	0.76
ATG10	ATG10 autophagy related 10 homolog	No	0.71	3.65	0.19	−0.25
CHES1	checkpoint suppressor 1	No	2.63	3.48	0.19	0.16
LOC92345	hypothetical protein BC008207	No	3.04	0.72	0.19	0.15
MSRB3	methionine sulfoxide reductase B3	No	0.86	3.17	0.19	0.20
ITFG1	integrin alpha FG-GAP repeat containing 1	No	1.76	3.48	0.18	0.40
KPNA1	karyopherin alpha 1 (importin alpha 5)	No	2.15	3.43	0.18	0.16
LOC652614	similar to HLA class I, A-11 alpha chain precursor	No	3.26	absent	0.16	absent
SIRT1	sirtuin (silent mating type information regulation 2 homolog)	No	3.98	2.76	0.16	0.13
CNKSR1	connector enhancer of kinase suppressor of Ras 1	No	1.11	3.41	0.16	0.06
LOC390933	similar to hypothetical protein	No	3.39	absent	0.15	absent
NPB	neuropeptide B	No	3.13	2.20	0.15	0.18
ARHGEF7	Rho guanine nucleotide exchange factor 7	No	3.27	2.82	0.14	0.16
XRRA1	X-ray radiation resistance associated 1	No	0.33	3.30	0.13	0.23
FAM21C	family with sequence similarity 21, member C	No	3.18	2.45	0.13	0.08
MAP1B	microtubule-associated protein 1B	No	2.97	2.79	0.12	0.03
TM9SF2	transmembrane 9 superfamily member 2	No	3.46	2.48	0.12	0.14
TEX14	testis expressed sequence 14	No	3.28	0.67	0.11	0.03
METT10D	methyltransferase 10 domain containing	No	3.17	0.54	0.11	0.02
LOC644150	WAS/WASL interacting protein family, member 3	No	3.09	1.60	0.11	−0.03
LOC731250	hypothetical protein LOC731250	No	3.44	absent	0.10	absent
SPIN3	spindlin family, member 3	No	4.86	2.02	0.10	0.07
C8ORFK32	C8orfK32 protein	No	3.51	1.99	0.10	0.05
IPO7	importin 7	No	2.98	4.74	0.09	0.08
CASC4	cancer susceptibility candidate 4	No	4.03	1.98	0.09	0.29
LOC391845	similar to 40S ribosomal protein S15	No	3.45	2.59	0.09	0.19
SYT1	synaptotagmin I	Yes	4.00	3.52	0.08	0.15
MGC16169	hypothetical protein MGC16169	No	1.69	3.45	0.08	0.49
SPHK2	sphingosine kinase 2	No	3.66	1.16	0.08	0.02
HCCA2	HCCA2 protein	No	2.60	3.21	0.07	0.01
CYCS	cytochrome c, somatic	No	3.38	0.05	0.07	−0.02
SRRM2	serine/arginine repetitive matrix 2	No	3.82	0.73	0.07	−0.02
CAP2	CAP, adenylate cyclase-associated protein, 2	No	0.72	3.83	0.07	0.32
APBA3	amyloid beta (A4) precursor protein-binding, family A, member 3	No	1.85	3.68	0.07	0.04
AP1G1	adaptor-related protein complex 1, gamma 1 subunit	Yes	3.52	0.74	0.06	−0.13
CCND3	cyclin D3	No	1.56	3.64	0.06	−0.02
NET1	neuroepithelial cell transforming gene 1	No	3.40	1.94	0.06	0.00
DKFZP434A0131	DKFZp434A0131 protein	No	3.04	1.49	0.05	−0.07
CS	citrate synthase	No	3.35	2.47	0.05	0.01
CASK	calcium/calmodulin-dependent serine protein kinase	No	0.05	3.42	0.04	0.38
ARFGEF2	ADP-ribosylation factor guanine nucleotide-exchange factor 2	Yes	3.47	0.87	0.02	0.07
C11orf30	chromosome 11 open reading frame 30	No	3.06	2.25	0.02	0.21
PHKG2	phosphorylase kinase, gamma 2	No	3.50	3.83	0.02	−0.09
NUP50	nucleoporin 50 kDa	No	3.43	1.11	0.02	0.01
EYA3	eyes absent homolog 3	No	3.44	1.51	0.01	0.27
C14orf172	chromosome 14 open reading frame 172	No	3.10	3.17	−0.04	−0.10
DOCK3	dedicator of cytokinesis 3	No	1.58	3.83	−0.06	0.12
GPSN2	glycoprotein, synaptic 2	Yes	1.78	4.61	−0.08	−0.05
APBA2	amyloid beta precursor protein-binding, family A, member 2	No	2.79	3.25	−0.09	−0.10
TAGLN3	transgelin 3	No	1.27	4.16	−0.09	−0.16
PTRF	polymerase I and transcript release factor	No	3.08	1.56	−0.09	−0.17
HSPBP1	hsp70-interacting protein	No	2.15	3.18	−0.10	−0.18
RPL3	ribosomal protein L3	No	2.36	3.45	−0.10	−0.14
EPHB3	EPH receptor B3	No	3.59	3.09	−0.10	−0.16
ARFRP1	ADP-ribosylation factor related protein 1	Yes	3.12	2.26	−0.10	−0.12
DAB2IP	DAB2 interacting protein	No	1.30	3.34	−0.11	−0.15
GLT25D1	glycosyltransferase 25 domain containing 1	No	1.50	3.30	−0.11	−0.20
PHF2	PHD finger protein 2	No	0.42	3.57	−0.11	−0.25
C11orf2	chromosome 11 open reading frame2	No	2.62	3.58	−0.12	−0.15
ASPHD2	aspartate beta-hydroxylase domain containing 2	No	0.70	3.26	−0.12	−0.11
ARMC6	armadillo repeat containing 6	No	3.22	3.23	−0.12	−0.16
NCDN	neurochondrin	Yes	3.51	3.22	−0.12	−0.17
LOC731986	similar to cytochrome P450 monooxygenase CYP2T1	No	3.13	absent	−0.12	absent
KLP1	N-acetyltransferase 14	No	3.35	2.65	−0.12	−0.19
TBC1D10B	TBC1 domain family, member 10B	No	2.72	3.21	−0.12	−0.17
LOC51035	SAPK substrate protein 1	No	3.45	2.70	−0.13	−0.18
PACSIN2	protein kinase C and casein kinase substrate in neurons 2	No	4.51	1.36	−0.13	−0.10
CDC37	cell division cycle 37 homolog	No	1.96	3.79	−0.13	−0.17
SNX17	sorting nexin 17	Yes	3.14	3.48	−0.13	−0.21
GOLPH2	golgi phosphoprotein 2	Yes	1.27	3.70	−0.14	−0.17
SLC39A7	solute carrier family 39 (zinc transporter), member 7	No	3.08	2.96	−0.14	−0.19
PSAP	prosaposin (variant Gaucher disease and metachromatic leukodystrophy)	No	2.99	2.83	−0.14	−0.16
C11orf10	chromosome 11 open reading frame 10	No	3.78	3.47	−0.15	−0.18
TRIM41	tripartite motif-containing 41	No	2.97	3.74	−0.15	−0.19
POLG	polymerase (DNA directed), gamma	No	2.10	3.18	−0.15	−0.22
COBRA1	cofactor of BRCA1	No	1.98	3.74	−0.16	−0.23
SNAPC5	small nuclear RNA activating complex, polypeptide 5, 19 kDa	No	3.12	1.26	−0.16	−0.09
COASY	Coenzyme A synthase	No	2.30	4.16	−0.16	−0.22
STX10	syntaxin 10	Yes	3.85	2.17	−0.17	−0.19
LOC642209	similar to ribosomal protein L13a	No	3.53	absent	−0.18	absent
C9orf25	chromosome 9 open reading frame 25	No	2.95	2.51	−0.18	−0.18
RASSF1	Ras association (RalGDS/AF-6) domain family 1	No	3.05	1.83	−0.18	−0.17
AIP	aryl hydrocarbon receptor interacting protein	No	2.36	3.22	−0.18	−0.22
LOC391739	similar to chaperonin subunit 6a (zeta)	No	1.25	3.48	−0.19	−0.28
TTBK1	tau tubulin kinase 1	No	2.98	3.34	−0.20	−0.19
EEF1D	eukaryotic translation elongation factor 1 delta	No	2.30	3.57	−0.20	−0.19
LOC643276	similar to Nucleolar complex protein 2 homolog	No	3.20	3.25	−0.20	−0.23
LOC731083	similar to heterogeneous nuclear ribonucleoprotein L	No	3.14	absent	−0.20	absent
LOC727858	similar to Dynamin-1	No	3.26	1.97	−0.20	−0.35
LGICZ1	ligand-gated ion channel, zinc activated 1	No	3.09	2.20	−0.21	−0.05
LOC642129	similar to Nucleolar complex protein 2 homolog	No	3.16	absent	−0.21	absent
LOC442157	similar to heterogeneous nuclear ribonucleoprotein L	No	2.98	2.39	−0.21	−0.23
ENO2	enolase 2 (gamma, neuronal)	No	2.75	3.32	−0.22	−0.26
EIF2C1	eukaryotic translation initiation factor 2C, 1	No	2.49	3.58	−0.22	−0.25
PHF19	PHD finger protein 19	No	2.46	3.18	−0.22	−0.24
ACTL6B	actin-like 6B	No	3.24	2.86	−0.22	−0.25
BUD13	BUD13 homolog	No	3.53	3.34	−0.22	−0.22
SV2A	synaptic vesicle glycoprotein 2A	Yes	2.98	2.80	−0.23	−0.26
PSMD2	proteasome 26S subunit, non-ATPase, 2	No	3.15	3.09	−0.23	−0.27
LOC728764	similar to Dynamin-1	No	3.68	2.02	−0.24	−0.32
HIRA	HIR histone cell cycle regulation defective homolog A	No	3.34	1.13	−0.25	−0.12
ZBTB22	zinc finger and BTB domain containing 22	No	3.48	3.29	−0.27	−0.30
LOC728791	similar to 40S ribosomal protein S10	No	1.82	4.81	−0.27	−0.38
WBSCR1	eukaryotic translation initiation factor 4H	No	3.13	3.09	−0.34	−0.37
LOC652489	similar to SMT3 suppressor of mif two 3 homolog 2 pseudogene	No	2.23	3.90	−0.34	−0.42
LOC651008	similar to mitogen-activated protein kinase kinase 3 isoform A	No	3.88	absent	−0.43	absent
CNGB1	cyclic nucleotide gated channel beta 1	No	3.33	0.18	−0.44	0.05
LOC389179	similar to Elongation FacTor family member4	No	3.40	3.26	−0.45	−0.51
LOC441891	similar to CG17293-PA	No	2.27	3.43	−0.47	−0.54
LOC442181	similar to 40S ribosomal protein SA	No	0.74	4.95	−0.48	−0.47
LOC347411	similar to brain protein 44-like	No	2.83	3.61	−0.54	−0.36
SLC25A2	solute carrier family 25 (ornithine transporter) member 2	No	0.04	3.62	−0.59	−0.02
LOC126170	similar to peptidylprolyl isomerase A isoform 1	No	3.03	1.32	−0.73	−0.50

1: Reads aligned to RefSeq transcript database; 2: Not detected in transcript aligned control sample reads; 3: Absent from genome database.

Genes with the largest increase in expression in SCZ samples were the ISL2 transcription factor, LIM/homeodomain, >800% in genome and transcript alignments; AMME complex, gene 1, 580% in transcript alignments; 3-hydroxy-3-methylglutaryl-Coenzyme A synthase, 372% in transcript alignments; kelch domain containing 1, 360% in transcript alignments; interphotoreceptor matrix proteoglycan 2, 321% in transcript alignments; and NK2 transcription factor related, locus 3, 320% in genome alignments. Genes with the largest decrease in expression in SCZ samples were the solute carrier family 25 (ornithine transporter), member 2, 59% in transcript alignments and LOC126170, similar to peptidylprolyl isomerase A isoform 1, 73% in transcript alignments.

An SCZ candidate gene showing differential expression by both measures was the gamma-aminobutyric acid (GABA)-mediated neuroinhibitory receptor, GABRA1 (increased expression in SCZ by 52% [transcript alignment] and 46% [genome alignment], Table 3). Eleven other genes involved in GABA neurotransmission showed non-significant increases in expression: GABRA2 (59%), GABRA4 (72%), GABRR1 (69%), GABRB1 (168%), GABRG2 (42%), GABRE (35%), GABRB2 (33%), GABBR2 (27%), GABARAPL2 (35%), GABRA6 (14%) and GABRB3 (8%). Four GABAergic genes showed non-significant decreases in expression: GABBR1 (22%), GABRG3 (54%), GABRR2 (26%) and SLC6A1 (GAT-1; 16%).

GO annotation of genes with significantly altered expression revealed over-representation of membrane associated genes (Χ^2^ test, p<0.01), genes involved in Zinc binding or transport (p<0.02), regulation of transcription (p<e^−6^), Golgi apparatus (p<e^−6^) and vesicle-mediated transport (p<0.01). 13 of 20 genes involved in vesicular transport or Golgi apparatus were up-regulated.

**Figure 10:**
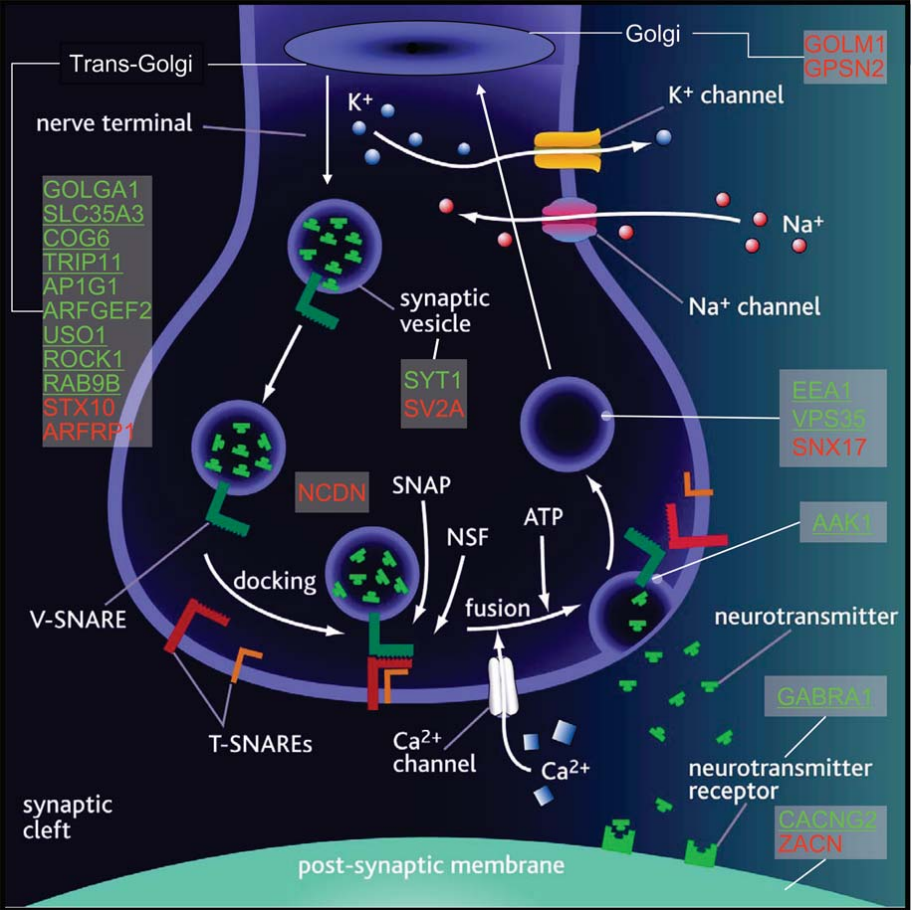
Cartoon illustrating functions and/or synaptic locations of 23 proteins corresponding to genes with altered expression in SCZ. 15 genes were upregulated (green), whereas 8 were downregulated (red). Underlined genes had >30% change in expression. Two genes involved in transport from the endoplasmic reticulum to the Golgi (GOLM1 and GPSN2) were downregulated, ten involved in transport from the trans-Golgi network to the synaptic vesicle were upregulated (GOLGA1, SLC35A3, COG6, TRIP11, AP1G1, ARFGEF2, USO1, ROCK1, RAB9B and VPS35) and two were downregulated (STX10 and ARFRP1), two genes involved with synaptic vesicle exocytosis (EEA1 and SYT1) were upregulated and two were downregulated (SV2A and NCDN), one gene involved in receptor-mediated endocytosis was upregulated (AAK1) and one involved in retrograde transport back to the Golgi apparatus was downregulated (SNX17). In addition, three post-synaptic membrane genes showed altered expression: GABRA1 (upregulated), ZACN (downregulated) and CACNG2 (upregulated).

Further annotation of these 20 genes revealed up-regulation of:

▪ Nine genes encoding proteins involved in transport from the trans-Golgi network to the synaptic vesicle (golgi autoantigen, subfamily a, member 1, **GOLGA1**, 28% in transcript alignments, 14% in genome alignments; solute carrier family 35 (UDP-N-acetylglucosamine transporter), member A3, **SLC35A3**, 69% in transcript alignments, 18% in genome alignments; component of oligomeric golgi complex 6, **COG6**, 30% in transcript alignments, 37% in genome alignments; thyroid hormone receptor interactor 11, **TRIP11**, 48% in transcript alignments, 23% in genome alignments; adaptor-related protein complex 1, gamma 1 subunit, **AP1G1**, 6% in transcript alignments,−13% in genome alignments; ADP-ribosylation factor guanine nucleotide-exchange factor 2, **ARFGEF2**, 2% in transcript alignments,7% in genome alignments; vesicle docking protein p115, **USO1**, 36% in transcript alignments, 25% in genome alignments; Rho-associated, coiled-coil containing protein kinase 1, **ROCK1**, 85% in transcript alignments, 60% in genome alignments; **RAB9B**, 35% in transcript alignments, 23% in genome alignments and vacuolar protein sorting 35 homolog,
▪ **VPS35** (52% in transcript alignments, 31% in genome alignments), which is involved in retrograde transport to the Golgi apparatus,
▪ Early endosome antigen 1, 162 kD, **EEA1** (88% in transcript alignments, −1% in genome alignments), which is involved in homotypic fusion of early endosomes,
▪ Synaptotagmin I, **SYT1** (8% in transcript alignments, 15% in genome alignments), which is involved with synaptic vesicle exocytosis, and
▪ AP2 associated kinase 1, **AAK1** (64% in transcript alignments, 34% in genome alignments), which is involved in receptor-mediated endocytosis (Fig. 10, Table 3).

Down-regulated transcripts corresponded to:

▪ Two genes involved in transport from the trans-Golgi network to the synaptic vesicle (syntaxin 10, **STX10**, −17% in transcript alignments, −19% in genome alignments and ADP-ribosylation factor related protein 1, **ARFRP1**, −10% in transcript alignments, −12% in genome alignments),
▪ Two genes involved in transport from the endoplasmic reticulum to the Golgi (golgi phosphoprotein 2, **GOLM1**, −14% in transcript alignments, −17% in genome alignments and synaptic glycoprotein 2, **GPSN2**, −8% in transcript alignments, −5% in genome alignments),
▪ Two genes involved with synaptic vesicle exocytosis (synaptic vesicle glycoprotein 2A, **SV2A**, −23% in transcript alignments, −26% in genome alignments and neurochondrin, **NCDN**, −12% in transcript alignments, −17% in genome alignments), and
▪ Sorting nexin 17, **SNX17** (−13% in transcript alignments, −21% in genome alignments) which is involved in retrograde transport back to the Golgi apparatus (Fig. 10, Table 3).

In addition, three post-synaptic membrane genes showed altered expression: **GABRA1** (see above), **ZACN** (ligand-gated ion channel, zinc activated 1; down-regulated 21% in transcript alignments and 5% in genome alignments) and **CACNG2** (calcium channel, voltage-dependent, gamma subunit 2; up-regulated 169% in transcript alignments and 10% in genome alignments) (Fig 10, Table 3).

### Identification of Novel Splice Isoforms

**Figure 11:**
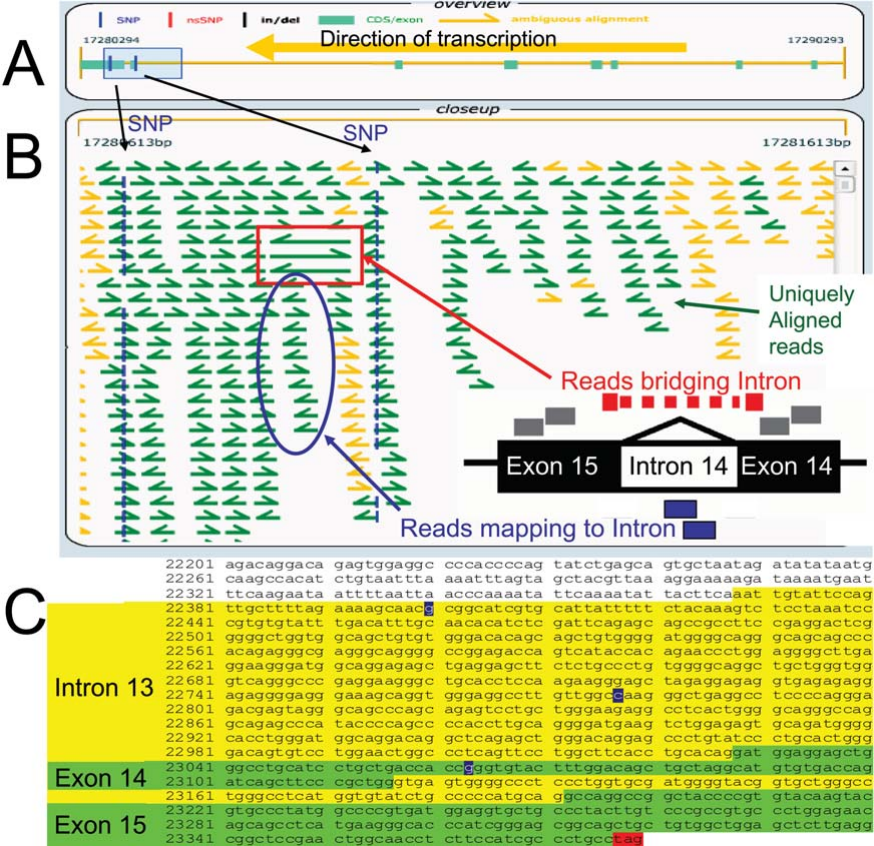
Novel alternative splicing of the *PRODH* locus. A. Exons (green boxes) and introns (yellow lines) of the *PRODH* locus are shown. A 100 bp shaded box covers part of intron 13, intron14 and exon 14 and part of exon 15. Two sSNPs are illustrated by vertical blue hash marks. B. Alignments of SBS reads to the introns and exons within the 1000 bp shaded box are shown. Sequence reads are shown as arrows pointing in the direction of their orientation relative to the genomic reference. Yellow arrows represent sequence reads that map to more than one region on the genomic reference, and green arrows represent uniquely mapping reads. Three cDNA reads that omit intron 14 and contain exon 14 and 15 sequences are highlighted. Also highlighted are cDNA reads that map within intron 14. C. The sequence of the PRODH genomic region shown in B. Green highlights represent exonic nucleotides with aligned sequence reads and yellow represent intronic nucleotides with aligned sequence reads. Reads aligning uniquely to intron 14 (75 nts) indicate the existence of an alternative splice isoform that reads through this intron. Similarly, the 3′ region of intron 13 appears to be included in a novel splice isoform(s).

10,022 genes exhibited ≥20% more reads aligned to genomic loci than to the corresponding, annotated transcripts, evidence for novel, unannotated exons. Putative use of alternative 5′ and 3′ terminal exons, novel internal exons and read-through of introns were all detected [57]. A SCZ susceptibility locus that exhibited intron-read-through in cerebellar mRNA samples was proline oxidase (*PRODH*, EC 1.5.99.8, SCZ4): 421 cerebellar cortex 41 reads aligned to the sole annotated *PRODH* transcript, while 838 aligned to the genomic locus. 33 of the latter mapped to intron 14 (nucleotides 17280876–17280950 on Chromosome 22), resulting in insertion of 25 new, in-frame amino acids adjacent to the N-terminus (Fig. 11). In addition to reads mapping within this intron, reads showing splicing out of the intron were also observed (Fig. 11). A further 112 reads aligned to 660 nucleotides of intron 13, generating an alternative N-terminus and premature stop codon (Fig. 11). Presence of these intronic *PRODH* sequences was observed in all cerebellum mRNA samples and is supported by independently isolated Sanger EST sequences (Genbank accession numbers CN362766, AA300535, AA322439, CD671570, CN362770).

As noted above, the presence of unannotated exons and novel splice isoforms appeared to explain disparity in the magnitude of gene expression change between cases and controls when using genome- and transcript-alignments. Dedicator of cytokinesis 3 (DOCK3), for example, was significantly up-regulated in SCZ using genome alignments (225 reads/million in cases versus 200 reads per million in controls, −log10(p-value) = 3.82) but non-significantly decreased using transcript alignments (138 reads/million in cases versus 147 reads per million in controls). Inspection of genomic read alignments revealed the existence of several, putative, unannotated exons. For example 26 reads in SCZ case 5S and 17 reads in SCZ case 5 aligned uniquely to DOCK3 intronic sequences corresponding to nucleotides 5,130,7158–51307328 on Chromosome 3 (data not shown).

## Discussion

As the first stage of a genomic convergence analysis of SCZ, we sought gene expression changes between post-mortem cerebellar cortices of 14 patients and 6 controls using shotgun, clonal mRNA sequencing, together with a rigorous and systematic analysis approach. Cerebellum was chosen for study since it has been shown to be affected in SCZ: Patients exhibit impaired sensory-motor integration, reduced cerebellar volume and grey matter, increased cerebellar blood flow and glucose uptake, both at rest and following cognitive challenge, greatest approximate conditional likelihood score in cerebellar vermis, and gene expression changes in the lateral hemispheres of the cerebellar cortex consistent with decreased GABAergic and increased glutamatergic neurotransmission [32], [51], [52], [58]–[66]. In addition, the simple organization and lack of perturbation by antipsychotic medication of cerebellar cortical circuits relative to other brain regions affected by SCZ was appealing for the current study. Specifically, granule cells, which are important in processing sensory information, are by far the most abundant cerebellar glutamatergic neuron, comprising the majority of neurons in the brain [67].

Cases and controls were all male and were matched reasonably well for age, race, cause of death and RNA quality metrics. Since only cases had received psychotropic medication, this variable could not be matched. There is currently considerable excitement about stratification of patients with SCZ on the basis of clinical or neuroimaging endophenotypes [20]. Unfortunately, that was not possible in this series.

Approximately 22 million random, cDNA sequence tags were generated from each of 20 samples, providing the deepest mRNA coverage reported to date and providing an unparalleled assessment of the transcriptional complexity of the human cerebellar cortex. Sequences did not align to 1879 viral genomes, offering negative evidence of chronic viral etiology for SCZ in these patients. 85% of annotated transcripts were expressed (based on molecular counting, Table 2), of which 63% were detected at a level of at least 1-fold coverage (equivalent to 56 reads per million or 1.1 copies per million). This pervasive transcription accords with other recent studies [32], [44], [56], [68]. In contrast to 3′ end tag sequencing, random-primed mRNA sequencing provided good coverage of coding domains (Fig. 1B), allowing assessment of exon utilization in cerebellar cortex. Alternative splicing is thought to generate more than 5 transcripts per human locus, most of which have not yet been annotated [56]. 85% of loci expressed at a level of at least 1-fold coverage showed evidence for utilization of unannotated exons, based on excess genomic read alignments. Proline oxidase, for example, is a very well studied gene that appeared to utilize hitherto unannotated exons in human cerebellum (Fig. 11). For this reason, sequences were aligned both to a reference transcript database (for estimation of expression of well-annotated transcripts) and to the human genome (for estimation of total expression of all exons, whether annotated or not). With additional improvements in library preparation and analysis software it should be possible to measure digital gene expression on an exon-by-exon basis in a strand-specific manner [31]–[50], [69]. The number of transcripts with aligned reads declined greater than exponentially as a function of level of expression (Fig 1B). As a result, 29 million aligned reads provided an impressive linear dynamic range (4.37×log10, Fig. 2). Despite lower read lengths and quality scores in this study (Table 2) than currently obtained with the Illumina GA II instrument, only 9% of reads mapped non-uniquely. Finally, technical imprecision of DTE measurements was paltry (CV 3.4%). In summary, our technical assessment indicated deep mRNA sequencing to be a powerful tool for digital gene expression and empiric annotation of transcribed elements in genomes, in accord with a rapidly growing literature [31]–[50], [69].

Gratifyingly, digital transcript expression values, expressed as aligned read frequencies, were amenable to very similar data transformation, quality control, pattern discovery, row-by-row modeling and annotation analyses as array hybridization datasets. This enabled direct comparison of digital transcript expression and array hybridization data metrics (Figs. 4–9). Like array hybridization signals, aligned read frequencies benefited from log transformation as evidenced by improved Mahalanobis distances, overlaid kernel density estimates, univariate distribution results, and unsupervised principal component analysis. Aligned read frequencies had greater correlation coefficients in pair wise sample comparisons than array hybridization, and much greater ability to distinguish SCZ cases from controls, as evaluated by unsupervised principal component analysis (PCA), Ward hierarchical clustering of Pearson product-moment correlations of read frequencies, and the magnitude of the component of variance attributable to diagnosis. DTE was clearly better than array hybridization for PCA-based segregation of cases and controls. Read frequencies based upon alignment to a reference transcript database and to the human genome were almost indistinguishable by these metrics. Partitioning of variance components of clinical, sample and experimental metadata revealed the largest components of array hybridization variability to be metrics of mRNA quality, in accord with recent studies of post-mortem brain tissue [70]. DTE was much less influenced by RNA quality. Non-diagnosis DTE variability included year of sequence generation, sequencing instrument variation and cause of death (Fig. 8). This is not surprising since substantial improvements occurred in sequencing instrument specifications, reagents and base-calling software during the project. Given continued, rapid evolution of generation II sequencing technologies, it is important to keep instrument specification, reagents and base-calling software relatively stable during mRNA sequencing projects to minimize non-hypothesis-related variability. Importantly, variance decomposition allowed incorporation of large clinical, sample and experimental effects as fixed effects in analyses of variance, minimizing detection of gene expression changes associated with confounding variables. Incorporation of fixed effects in array hybridization, but not DTE, analyses of variance resulted in no expression changes meeting significance cutoffs. Coefficients of correlation between aligned read frequencies, adjusted for transcript length, and array hybridization signals were 0.35 for all values and 0.46 for fold-change in genes with significant differences in expression. These values are somewhat lower than similar comparisons between array hybridization platforms and are probably reflective of the exacting RNA source [71]. It should be noted that aligned read frequencies correlate very well with quantitative PCR results [57], [72]. In conclusion, mRNA sequencing appears to be superior to array hybridization for gene expression analysis and is amenable to standard statistical procedures for quality assessment and gene expression analysis.

Expression of 215 genes differed significantly between cases and controls in post-mortem cerebellar cortex by analysis of variance (Table 3). Of these, 88 were identified in genome-aligned reads and 152 in transcript-aligned reads. Only 25 genes were common to both alignments, but 96% of the 215 had congruent direction of change and correlation between genome- and transcript-aligned fold-change was 0.73. Major contributors to disparity between alignments appeared to be detection of unannotated exons in genome alignments and forced mis-alignments in incomplete transcript datasets. The latter may largely be avoided by removal of non-unique alignments from calculations. As RefSeq transcript becomes more complete, this disparity should decrease. In the interim, it is wise to align both to a well-annotated transcript dataset and to the genome.

17 of the 215 genes with altered cerebellar expression have previously shown changes in SCZ in the dorsolateral prefrontal cortex or superior temporal gyrus (APBA2, BTG1, CACNG2, CAP2, DKFZP434A0131, GABRA1, GOLGA1, HSPBP1, KIAA0256, KPNA1, RPL3, SLC35A3, SLC39A7, SRRM2, TCF4, ZNF148 and ZNF195). Despite substantial differences in gene expression in cerebellar cortex and cerebral cortex [73], 13 genes were congruent with the direction of change reported previously. Three genes involved in GABAergic neurotransmission (GABRA6, GABRB3 and SLC6A1) showed changes in cerebellar cortex in SCZ that agreed with a previous report [52]. In the context of cerebellar cortical function in SCZ, alteration in expression of genes may be causal, consequential, compensatory, or the result of confounding factors. Because the substantial heritability of schizophrenia appears to be polygenic [1]–[5], expression differences might reflect SCZ-associated nucleotide variants that alter expression in cis (e.g. eSNPs). Indeed, seven of these genes have previously shown association with SCZ in genetic association studies (CACNG2, GABRA1, GPSN2, HIRA, PSAP, RANBP5 and TCF4) [74]. Alternatively, expression differences may represent compensatory changes in pathways and networks. GO annotation of genes with altered expression revealed over-representation of membrane association, Zinc binding or transport, regulation of transcription, Golgi apparatus and vesicle-mediated transport.

Most striking were 23 genes involved in presynaptic vesicular transport / Golgi apparatus or post-synaptic neurotransmission, 15 of which were up-regulated and 8 down-regulated. Up-regulated genes included nine involved in transport from the trans-Golgi network to the synaptic vesicle (golgi autoantigen A1, UDP-N-acetylglucosamine transporter (SLC35A3), component of oligomeric golgi complex 6, thyroid hormone receptor interactor 11, adaptor-related protein complex 1G1, ADP-ribosylation factor guanine nucleotide-exchange factor 2, vesicle docking protein p115, Rho-associated, coiled-coil containing protein kinase 1 (ROCK1) and RAB9B), one involved in synaptic vesicle exocytosis (synaptotagmin I), one involved in clathrin-mediated endocytosis (AP2 associated kinase 1 (AAK1)), one involved in homotypic fusion of early endosomes (early endosome antigen 1 (EEA1), and one involved in retrograde transport to the Golgi apparatus (vacuolar protein sorting 35 (VPS35)). Down-regulated genes included two involved in transport from the trans-Golgi network to the synaptic vesicle (syntaxin 10 and ADP-ribosylation factor related protein 1), two involved with synaptic vesicle exocytosis (synaptic vesicle glycoprotein 2A and neurochondrin), two involved in transport from the endoplasmic reticulum to the Golgi (golgi phosphoprotein 2 and synaptic glycoprotein 2) and one involved in retrograde transport to the Golgi apparatus (sorting nexin 17) (Fig. 10, Table 3). Up-regulated post-synaptic membrane genes included GABRA1 (see above) and CACNG2 (calcium channel, voltage-dependent, gamma subunit 2). Several other genes involved in GABAergic neurotransmission showed non-significant changes in expression in cerebellar cortex in SCZ, in agreement with previous reports [52]. One down-regulated post-synaptic membrane gene was ligand-gated ion channel, zinc activated 1. Most of the up-regulated genes, but none of the down-regulated genes in this set, had change in expression of greater than 30%. The largest alterations were CACNG2 (+169%), EEA1 (+88%), ROCK1 (+85%), SLC35A3 (+69%), AAK1 (64%) and GABRA1 and VPS35 (both +52%). Several previous studies have shown decreased expression of genes involved in presynaptic vesicular transport in the dorsolateral prefrontal cortex and hippocampus in SCZ [75]–[78]. While the molecular determinants of synaptic vesicular transport have been intensively studied, it is not yet possible to integrate these transcriptional changes into a single biological outcome. Isolated elevation of AAK1, for example, should decrease neurotransmitter endocytosis [79], while elevated EEA1 should increase early endosomal fusion [80]. However, activity of most of these proteins is dependent either upon post-translational modification or presence of interacting proteins. Furthermore, there is likely to be heterogeneous expression of these proteins among cerebellar cortical neurons. Nevertheless, it appears clear that presynaptic vesicular transport, Golgi function and GABAergic neurotransmission are perturbed in cerebellar cortex in SCZ, in agreement with previous analyses of gene expression in post-mortem brain in SCZ [28], [52], [75] and genetic associations of nucleotide variants in synaptic vesicular transport genes with SCZ (namely CACNG2, COG2, STX1A, SNAP29, MUTED, NRG1, PLDN, NRG3, HOMER3, RTN4R, NRG2, RTN4, CPLX2, BLOC1S1, SYNJ1, HOMER1, CLINT1, SYP, BLOC1S2, SYN3, SYT11, SYNGR1, SNAPAP, STX7, BLOC1S3, DISC1, SNAP25, DLG4, CPLX1, CNO, SYN2, DTNBP1 and DAOA) [74]. Replication of alterations in presynaptic vesicular transport and Golgi function, particularly as affecting GABAergic neurotransmission, in additional cases or other brain regions–such as prefrontal cortex–would substantiate novel molecular mechanisms underpinning SCZ and establish novel targets for therapeutic intervention.

The current study reports the first phase of a genomic convergence analysis of SCZ, which is intended to integrate linkage and expression studies. In a subsequent manuscript, we will present results of expressed nucleotide variants that differ in allele frequency between cases and controls, and integrate nucleotide variant and expression analyses. In addition, a recent study has shown substantial variation in alternative splice isoform expression and alternative polyadenylation in cerebellar cortex between normal individuals [57], and it will be interesting to ascertain whether specific examples of alternative isoform expression show association with SCZ. Confirmation of causal components of the molecular basis of SCZ is anticipated to have significant impact on clinical practice, particularly with respect to timely diagnosis and prognostic or therapeutic categorization [12], [19].

## Materials and Methods

### Sample preparation

was as previously described [51]. Anonymized cerebellar samples were provided from the Maryland Brain Collection with permission from the Maryland Brain Collection Steering Committee. Permission for the study of brain tissues was provided by families post-mortem in accordance with the guidelines of the Uniform Anatomical Gift Act. Fourteen samples were from patients with a diagnosis of SCZ according to DSM-IV criteria and six were controls. Cortical areas corresponding to crus I/VIIa of cerebellar hemispheres were dissected at −20°C and frozen at −80°C, as described [52]. The average pH of the samples was 6.6±0.17. Cerebellum specimens were obtained according to NIH guidelines for confidentially and privacy. Genomic DNA and total RNA were isolated from samples using standard techniques (Qiagen, Valencia, CA).

### Sequencing-by-synthesis

Cerebellum samples were sequenced using Illumina Genome Analyzers with modifications for mRNA samples [39], [42], [43], [52], [57]: Following quality assessment using a Bioanalyzer 2100 (Agilent Inc., Santa Clara, CA; Table 1), poly A+ RNA was isolated from 5–10 µg total RNA by two rounds of oligo-dT selection (Invitrogen Inc., Santa Clara, CA). mRNA was annealed to high concentrations of random hexamers and reverse transcribed. Following second strand synthesis, end repair, and A-tailing, adapters complementary to sequencing primers were ligated to cDNA fragment ends. Resultant cDNA libraries were size fractionated on agarose gels, 200 bp fragments excised, and amplified by 15 cycles of polymerase chain reaction. Following quality assessment using a Bioanalyzer 2100, single-stranded cDNA-adapter fragments were randomly annealed to the surface of a flow cell in a cluster station (Illumina Inc., San Diego, CA) via primers complementary to the adapters and incubated under conditions fostering annealing of the ends of cDNA-adapter fragments to adjacent complementary primers. Primers with annealed cDNA-adapter fragments were extended with DNA polymerase and unlabelled dNTPs in a solid-phase “bridge amplification”. Resultant double-stranded products were denatured, yielding 2 single stranded fragments. The latter steps were repeated for 35 cycles, generating ∼40 million clusters of clonal amplicons. Subsequently, 32–36 cycles of sequencing-by-synthesis chemistry were performed in Illumina Genome Analyzer instruments with 4 dNTPs featuring cleavable dyes and reversible terminators. Following each base extension, 4 images (one for each nucleotide) are taken upon laser excitation. Incorporation of the next base occurred after removal of the blocked 3′ terminus and fluorescent tag of the previously incorporated nucleotide. Sequences were retained if of high quality (defined as those passing the default quality filtering parameters used in the Illumina GA Pipeline GERALD stage, i.e. clusters with intensities greater than 0.6-times the average of the highest and the sum of the two highest intensities for the first 12 cycles). Furthermore, sequencing runs with poor average nucleotide quality score graphs (associated with a minority of reads passing the default quality filtering parameters) were discarded.

### Read Alignment-based gene expression profiling

High-quality reads were aligned to the human genome, Build 36.2, RefSeq transcript database, Release 22 [81], Unigene Hs, build 215 [82] using the algorithm GMAP [55] and the software system Alpheus® [48], with adjustments for short SBS reads (oligomer overlap interval = 3 nt, identities ≥34/36 or 94%). A read was denoted ‘aligned’ to a locus if its sequence alignment to the genomic reference sequence (NCBI build 36.2) fell within the boundaries of the locus coordinates on the chromosome. Locus boundaries on the genome were defined by NCBI annotations, as reported through the Nucleotide database. Annotation data was downloaded on 2/2/2007 using the Batch Entrez query tools (http://www.ncbi.nlm.nih.gov/sites/batchentrez?dbNucleotide). Only the highest scoring alignment(s) was retained. Reads with a single best alignment or with equally good alignments to alternative transcripts of the same locus were considered uniquely aligned. Aligned read frequencies (per million aligned reads) were calculated for each sample and locus using Alpheus® [48].

Array-based gene expression profiling was performed using Affymetrix Human Genome U133 Plus 2.0 oligonucleotide arrays and standard procedures (Affymetrix, Santa Clara, CA). Array-based gene expression profiling of cerebellar cortex of unaffected individuals was performed using Affymetrix Human U95A oligonucleotide arrays as previously reported [83]. Primary image analysis was performed with Affymetrix GENECHIP 3.2. Images were scaled to an average hybridization intensity of 200, and the threshold for expression was an average hybridization intensity of ≥50.

### Statistical Analysis

Array hybridization signals and read frequencies were log10 transformed prior to evaluation of inter-sample differences. Overlaid kernel density estimates, univariate distribution results, Mahalanobis distances, correlation coefficients of pair wise sample comparisons, unsupervised principal component analysis (by Pearson product-moment correlation) and Ward hierarchical clustering of Pearson product-moment correlations of read frequencies were performed with JMP Genomics, Version 3.2 (SAS Institute, Cary, NC). Decomposition of principal components of variance of array signals and read frequencies, FDR corrected analysis of variance (with inclusion of the non-diagnosis components of variance discussed above as fixed effects) and chi squared comparisons of GO annotations were also performed with JMP Genomics, Version 3.2. Patient, sample, and experimental parameters examined to quantify sources of variability in measurements were age, alignment percentage, brain pH, cause of death, cluster station, diagnosis, library creator, number of reads, post-mortem interval, psychotropic medication, race, read length, read quality score, RNA isolation date, RNA integrity number, sample storage duration, sequencing instrument and year sequenced.
